# The Effectiveness of Conbercept Combined with Panretinal Photocoagulation vs. Panretinal Photocoagulation in the Treatment of Diabetic Retinopathy: A Meta-Analysis

**DOI:** 10.1155/2021/5591719

**Published:** 2021-05-08

**Authors:** Chao Huang, Haiqing Ji, Xuguang Han

**Affiliations:** ^1^Department of Ophthalmology, Qilu Hospital of Shandong University, Jinan, Shandong, China; ^2^Outpatient Department, Weifang Eye Hospital, Weifang, Shandong, China; ^3^Department of Ophthalmology, Jinan Second People's Hospital, Jinan, Shandong, China

## Abstract

**Objective:**

This meta-analysis aimed to compare the effect and safety of conbercept with panretinal photocoagulation (PRP) vs. PRP in the treatment of diabetic retinopathy (DR).

**Methods:**

Relevant studies were identified through systemic searches of PubMed, EMBASE, China National Knowledge Infrastructure, and Wanfang database up to December 2020. The results of conbercept and PRP in patients with DR were analyzed, including overall effectiveness, best corrected visual acuity, central macular thickness, and complications.

**Results:**

12 articles involving 1244 patients with DR were identified for this meta-analysis. The results of the meta-analysis showed that conbercept combined with PRP significantly increased the level of overall effectiveness and significantly reduced the central thickness of macula and the incidence of complications compared with the control group.

**Conclusions:**

Conbercept with PRP tended to be more effective than PRP alone in terms of functional outcomes for treating DR.

## 1. Introduction

Diabetic retinopathy (DR) is the most prevalent and severe ocular disorders and the major reason of adult blindness [1]. In USA, the overall 10-year incidence of retinopathy was 74%, and in people with retinopathy at baseline, 64% developed more severe retinopathy and 17% progressed to develop proliferative retinopathy [2, 3]. Due to the long-term hyperglycemia, the metabolism of capillary wall cells is disordered, and abnormal blood circulation will occur, which will affect the hardness and permeability of the retina [4]. Laser therapy has a relatively long history in the treatment of DR, and among them, the panretinal photocoagulation (PRP) was mainly applied. Based on the principle of photocoagulation, it could destroy the high-consumption retinal pigment epithelium tissue and make it scar, which can improve the state of retinal ischemia and effectively inhibit neovascularization [5]. However, drug therapy represented by antivascular endothelial growth factor (VEGF) drugs has emerged and become a hotspot in the treatment of DR in recent years.

Although laser technology can rapidly treat the lesion area, the anti-VEGF drugs can reduce the proliferation and leakage of tissue and blood vessels caused by laser surgery. It makes up for the shortcomings of laser surgery caused by temporary increase of the apparent membrane thickness and the decrease of visual acuity [6]. As an anti-VEGF fusion protein, conbercept can not only block the angiogenesis of pathological changes but also improve the blocking of ischemia and hypoxia, blood perfusion, and inflammatory cell expression [7, 8]. Conbercept ophthalmic injection is composed of humanized recombinant fusion protein, which can inhibit neovascularization [9]. In China, conbercept has been widely used as the first-line drug for the treatment of DR for nearly 5 years. Furthermore, some studies have reported the combination of PRP and conbercept intraocular injection could enhance the treatment effect and accelerate the recovery of vision.

To date, no systematic review has discussed the therapeutic effect and safety of conbercept versus PRP or conbercept alone in DR. Therefore, we performed this meta-analysis to quantify the efficacy and safety of conbercept and PRP in the treatment of DR.

## 2. Materials and Methods

A systematic search was performed to identify relevant studies of conbercept on the treatment of DR by using the following databases: PubMed, EMBASE, China National Knowledge Infrastructure, and Wanfang Data. The search included all published articles from August 2018 up to December 2020, with the following medical subject heading terms: (“conbercept” AND (“panretinal photocoagulation” OR “PRP”) AND (“Diabetic retinopathy” OR “DR”)). There were no language restrictions in the research. Inclusion criteria: (1) randomized controlled trials (RCTs); (2) the type of disease was DR; (3) the treatments were conbercept combined with PRP and PRP alone; (4) therapeutic efficacy indicators can be obtained, such as overall effective rate, best vision correction, central macular thickness and complications. Exclusion criteria: (1) repeated articles; (2) summary of the meeting, comments, letters, etc; (3) animal studies that existing meta-analysis and systematic evaluation.

### 2.1. Data Extraction

Two independent reviewers searched the articles, assessed the quality of trials, and extracted the following data with a standardized form: author's name, publication time, sample size, age, course of diabetes mellitus, and DR. The included articles bias was evaluated by the Cochrane Collaboration's RCT bias wind assessment tool. The modified Jadad scale was used to evaluate the quality of the included studies, which contains eight aspects: (1) was the research described as randomized? (2) Was the approach of randomization appropriate? (3) Was the research described as blinding? (4) Was the approach of blinding appropriate? (5) Was there a presentation of withdrawals and dropouts? (6) Was there a presentation of the inclusion/exclusion criteria? (7) Was the approach used to assess adverse effects described? (8) Was the approach of statistical analysis described? Studies receiving scores above five were considered of high quality.

### 2.2. Statistical Analysis

Stata software was used for all analyses. The total effective rate and complication effect were estimated using 95% CI and OR value. The effects of best corrected visual acuity and central macular thickness were estimated using weighted mean difference (WMD) and 95% CI. Random effects or fixed effects models were selected to estimate the total effects according to the heterogeneity test results. The *Q-*test and I^2^ test were used to estimate interstudy heterogeneity. When *P* > 0.1 and *I*^2^ ≤50%, the fixed-effect model was adopted. When *P* < 0.1 and *I*^2^ ≥50%, the random-effect model was adopted. Sensitivity analysis was used to evaluate the stability of the results. The Begger and Egger tests are used to assess publication bias. *P* < 0.05 was considered as statistically significant.

## 3. Results

**Figure 1:**
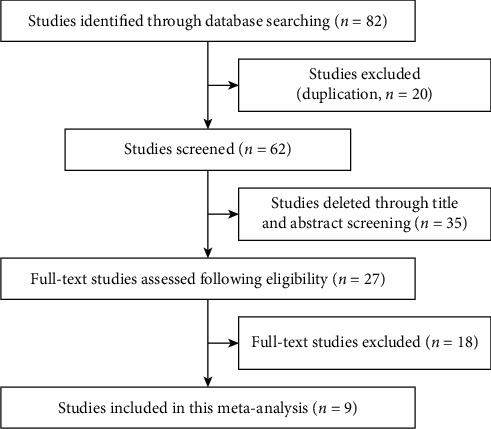
The process of selecting articles for the meta-analysis.

A total of 82 references were found after searching, and 20 duplicate references were eliminated. By reading the title and abstract, 35 unrelated references were excluded, and 27 full articles were read. According to the inclusion and exclusion criteria, 18 references were excluded, and 9 were finally included in this meta-analysis. 12 articles involving 1244 patients with DR were identified for this meta-analysis. The retrieval flow chart is shown in Figure 1. The basic information of the included studies is shown in Table 1. The patients' average age was over 50 years old, with a 6-year history of diabetes and 2-year history of DR.

**Figure 2:**
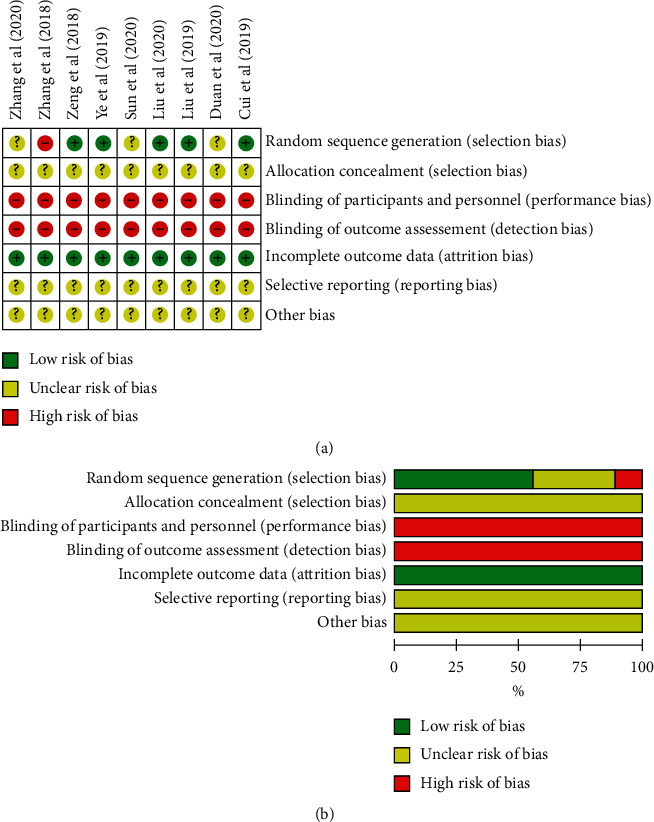
(a) The detailed characteristics of the included studies. (b) The articles' risk of bias evaluation results.

The articles' risk bias evaluation results are shown in Figure 2. The randomization method was described in 5 articles. All studies with complete data were described the setting of stratified seclusion and blindness. The article quality evaluation results are shown in Figure 1. The evaluation score of 5 articles was 5, and that of 4 articles was less than 5. The quality of the included article was medium.

**Figure 3:**
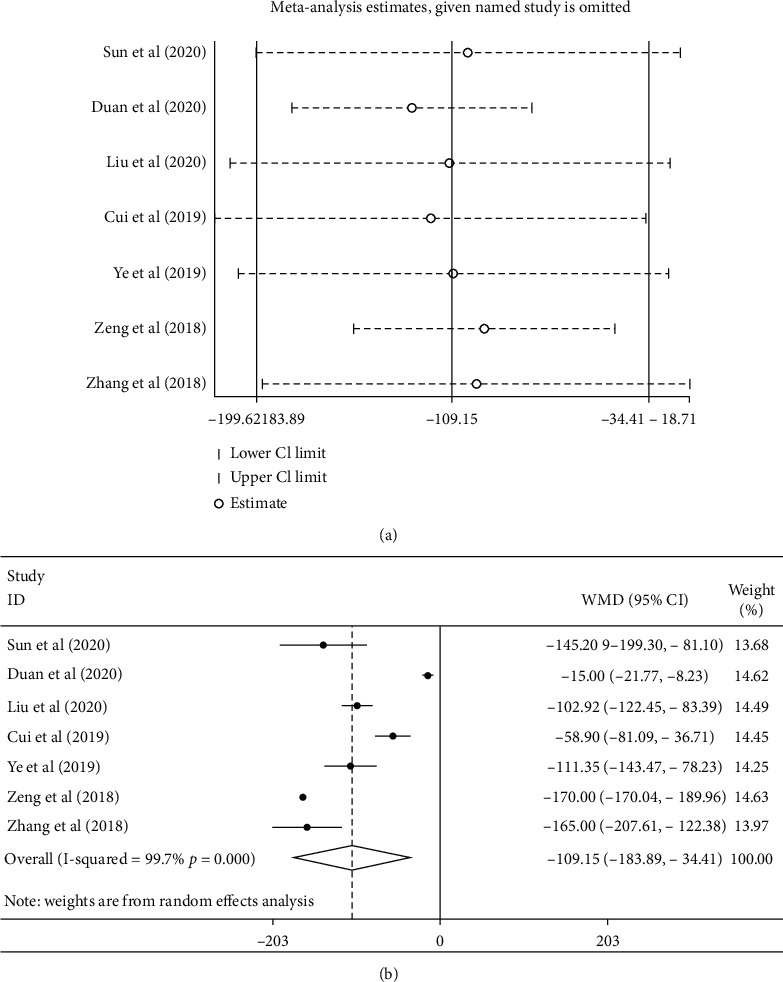
Forest plot of the overall effectiveness in conbercept treatment or compared with PRP that of DR.

5 articles reported the overall effectiveness of the two treatments. As shown in Figure 3, the fixed-effect model results showed that the overall efficiency of conbercept combined with PRP was higher than that of PRP alone, and the difference was statistically significant (OR = 6.11, 95% CI (3.36, 11.13), *P* < 0.0001; *I*^2^ = 0.0%, *P*=0.544). There was no heterogeneity among studies.

**Figure 4:**
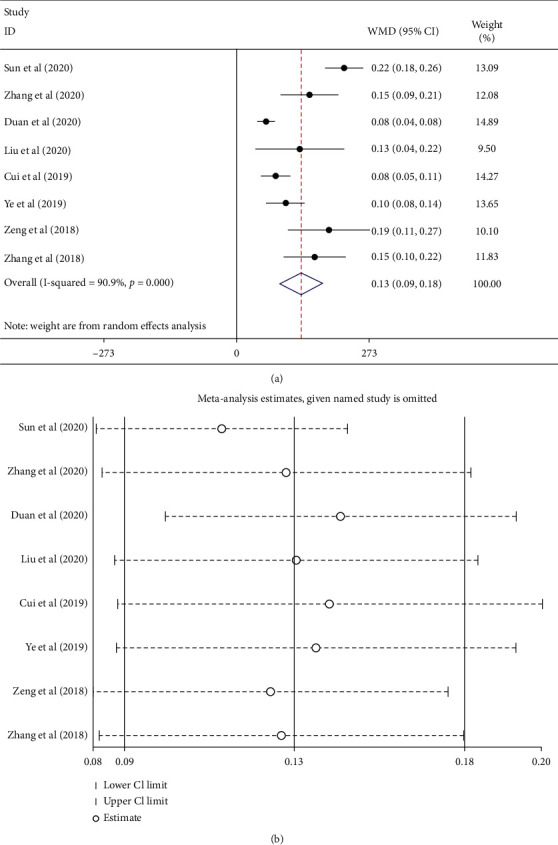
(a) Sensitivity analysis for conbercept combined with PRP was greater than that of PRP alone. (b) Forest plot of best corrected visual acuity of conbercept combined with PRP was greater than that of PRP alone.

8 articles reported changes in vision after two treatments. As shown in Figure 4(a), the random-effect model results showed that the best corrected visual acuity of conbercept combined with PRP was statistically significant greater than that of PRP alone (WMD = 0.13, 95% CI (0.09, 0.18), *P* < 0.0001; *I*^2^ = 90.0%, *P* < 0.0001), which indicated the heterogeneity existing among studies. Sensitivity analysis results are shown in Figure 4(b). The estimated total effect of each study was excluded successively within the range of 95% CI (0.09, 0.18), and the results were stable. No publication bias was detected by Begger test (*P*=0.174) and Egger test (*P*=0.05).

**Figure 5:**
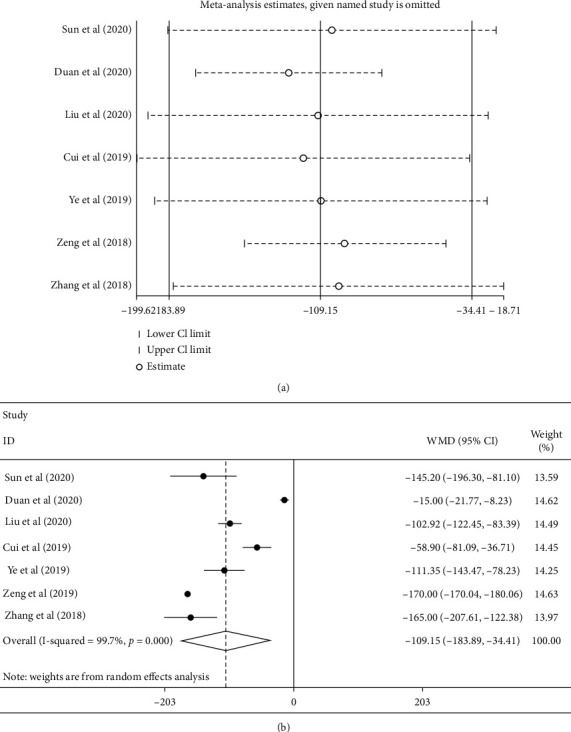
(a) Sensitivity analysis for conbercept combined with PRP was greater than that of PRP alone. (b) Forest plot of the central macular thickness in conbercept treatment or compared with PRP that of DR.

7 articles reported changes in central macular thickness after two treatments. As shown in Figure 5(a), the random effect model results indicated that the central thickness of the macular area was statistically significantly lower with conbercept combined with PRP than that with PRP alone (WMD = −109.15, 95% CI (−183.89, 34.14), *P*=0.0004; *I*^2^ = 99.7, *P* < 0.0001), which indicated the heterogeneity existing among studies. Sensitivity analysis results are shown in Figure 5(b). The estimated total effect of each study was excluded successively within the range of 95% CI (−183.89, 34.14), and the results were stable. No publication bias was detected by Begger test (*P*=0.548) and Egger test (*P*=0.174).

**Figure 6:**
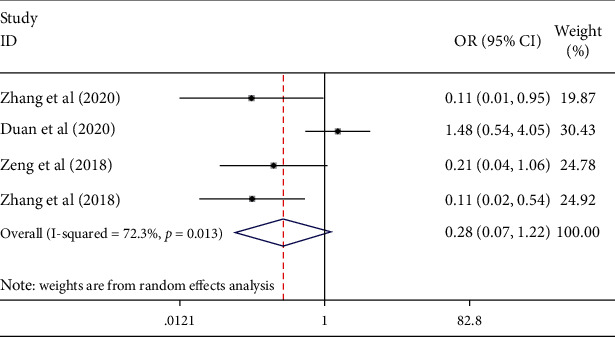
Forest plot of the complication rate of DR in conbercept treatment or combined with PRP.

4 articles reported the incidence of complications after two treatments. As shown in Figure 6, the random effect model results showed that the complication incidence of conbercept combined with PRP was lower than that of PRP alone, but with no statistically significant difference (OR = 0.28, 95% CI (0.07, 1.22), *P*=0.091; *I*^2^ = 72.3%, *P*=0.013), which indicated the moderate heterogeneity among studies.

## 4. Discussion

DR is the leading cause of severe vision loss in patients with diabetes worldwide [10]. The increase in the number of DR patients also puts a burden on the health care system [11]. In recent years, due to a variety of factors, the prevalence of diabetes continues to rise, and the number of DR patients accordingly increases, which has a serious impact on the quality of life of patients [12]. In the past four decades, PRP has been the standard treatment for DR, according to the American Academy of Ophthalmology's latest Diabetic Retinopathy Clinical Guidelines in 2019. It can induce the regression of neovascular and reduce the risk of severe vision loss [13]. However, He et al. and Soman et al. have indicated that PRP treatment may cause short-term macular edema [14, 15]. Recently, anti-VEGF drugs have been shown to effectively attenuate retinal neovascularization [16, 17], and intravitreal injection of anti-VEGF agents could also be used for DR. Furthermore, previous studies found that PRP combined with antivascular endothelial growth factor (VEGF) agents such as ranibizumab was more effective for neovascular regression than PRP alone [18–20]. Therefore, it is of great significance to find a therapeutic regimen with high safety and definite efficacy to improve the quality of life of DR patients.

Conbercept belongs to the group of recombinant decoy receptors to VEGF. Conbercept is a recombinant fusion protein which consists of the second Ig domain of VEGFR1 and the 3rd and 4th Ig domains of VEGFR2 combined with the constant region. PRP plus conbercept might be a better therapeutic strategy than PRP plus sub-Tenon's triamcinolone acetonide in treating DR at the proliferative stage [21]. For patients with macular edema, conbercept can better reduce the thickness of macular fovea, improve patients' visual acuity, and improve the treatment efficiency in a short period (3 months) than the control group, with reliable efficacy and good tolerability [22]. In addition, many scholars have compared the efficacy of conbercept with that of PRP [23–25]. However, there is still a lack of systematic classification, collection, and evaluation of these study data. Therefore, we performed this meta-analysis to quantify the effect and safety of conbercept and PRP in the treatment of DR.

In this study, 12 articles were selected strictly according to the inclusion criteria, and 1244 patients were included, over 50 years old, with a 6-year history of diabetes, and with a 2-year history of DR. The results fully indicate that PRP combined with intravitreal injection of conbercept can effectively promote the recovery of retinal thickness, improve the maximum corrected visual acuity, significantly shorten the time for symptom improvement, and reduce the incidence of complications in DR patients.

Intravitreal injection of conbercept combined with PRP for macular edema secondary to BRVO is effective, safe, and superior to PRP only. It also had a longer effective duration and less complication than intravitreal triamcinolone combined with laser photocoagulation [26]. There was an article shown that the combination of the two treatments can reduce the logMAR value of the patient's vision and can significantly improve the patient's hemodynamics, oxidative stress, and inflammatory factors [27]. It is consistent with the above conclusions that PRP and intravitreal injection of conbercept in the treatment of DR may effectively promote the recovery of retinal thickness and increase the maximum corrected visual acuity. At the same time, PRP treatment can significantly shorten the time of symptom improvements and reduce the incidence of complications and serum bFGF, IGF-1, and VEGF levels [28].

## 5. Conclusion

Our results demonstrated that the conbercept combined with PRP has better efficacy than that of PRP alone. Furthermore, patients in the combined group had significantly reduced the complications and central thickness of the macular area after the treatment than the PRP group. In contrast, patients in the combined group had increased overall effectiveness. Therefore, the combined therapy could be a potentially favorable treatment therapy for DR. However, there were still the following shortcomings: there were fewer included articles and they were all open studies, so the conclusions obtained in this paper still need to be verified in a large sample randomized, double-blind, controlled study. There is no explanation about blind method in the included studies, and there is a certain risk bias in the article. In future studies, we need higher quality studies to verify our conclusions.

## Figures and Tables

**Table 1:** Study baseline information was included.

Authors (year)	Stage	Intervention	Sampling	Age (year)	Diabetic duration	DR duration	Jadad scale	Follow-up
Sun et al. (2020)	Proliferative	0.1 ml conbercept + PRP	44	57.94 ± 8.87	8.39 ± 1.32	2.25 ± 0.40	3	1 month, 4 months
		PRP: 4-5 times, every other week	44	58.36 ± 8.49	8.47 ± 1.29	2.20 ± 0.41		

Zhang et al. (2020)	Proliferative	0.05 ml conbercept + PRP	24	58.63 ± 5.14	10.32 ± 1.27		3	1 month, 3 months
		PRP: 3-4 times, every other week	25	58.47 ± 5.10	10.42 ± 1.33			

Duan et al. (2020)	Proliferative	0.05 ml conbercept + PRP	100	62.3 ± 1.2	6.8 ± 1.0		3	1 month
		PRP: 3-4 times, every other week	100	60.0 ± 2.4	6.2 ± 1.2			

Liu et al. (2020)	Nonproliferative and proliferative	0.05 ml conbercept + PRP	56	56.27 ± 3.16			5	1 month
		PRP	56	57.86 ± 3.87				

Cui et al. (2019)	Proliferative	0.05 ml conbercept + PRP	32	57.2 ± 9.2	8.4 ± 3.9		5	1 month
		PRP: 3-4 times, every other week	32	59.0 ± 8.0	7.8 ± 4.5			

Ye et al. (2019)	Proliferative	0.05 ml conbercept + PRP	50	66.05 ± 10.23			5	3 months
		PRP: 3-4 times, every other week	50	65.84 ± 10.45				

Zeng et al. (2018)	Not applicable	0.05 ml conbercept + PRP	40	61.86 ± 12.77	7.61 ± 1.37		5	1 month
		PRP: 500–800 points per time, every other week	40	61.59 ± 12.82	7.57 ± 1.29			

Liu et al. (2019)	Nonproliferative	0.1 ml conbercept + PRP	48	58.34 ± 8.47	8.45 ± 1.27	2.18 ± 0.39	5	1 month
		PRP: 4 times, every other week	47	57.92 ± 8.85	8.37 ± 1.30	2.23 ± 0.38		

Zhang et al. (2018)	Not applicable	0.1 ml conbercept + PRP	31	50 ± 5	6 ± 3		4	3 months
		PRP: 500–800 points per time, every other 5d	31	49 ± 6	7 ± 3			

*Note.* DR, diabetic retinopathy; PRP, panretinal photocoagulation.

## Data Availability

The data used to support the findings of this study are available from the corresponding author upon request.
